# Numerical Simulation of Mass Transfer in Hollow Fiber Membrane Module for Membrane-Based Artificial Organs

**DOI:** 10.3390/membranes14030067

**Published:** 2024-03-10

**Authors:** Ziheng Wang, Shaofeng Xu, Yifan Yu, Wei Zhang, Xuechang Zhang

**Affiliations:** 1 School of Mechanical Engineering, Zhejiang Sci-Tech University, Hangzhou 310000, China; 202130605306@mails.zstu.edu.cn; 2 School of Mechatronics and Energy Engineering, NingboTech University, Ningbo 315000, China; 10925066@zju.edu.cn (W.Z.); xczhang@nit.zju.edu.cn (X.Z.); 3 School of Mechanical Engineering, Zhejiang University, Hangzhou 310000, China; 22225154@zju.edu.cn

**Keywords:** membrane-based artificial organs, hollow fiber membrane module, numerical simulation, mass transfer

## Abstract

The mass transfer behavior in a hollow fiber membrane module of membrane-based artificial organs (such as artificial liver or artificial kidney) were studied by numerical simulation. A new computational fluid dynamics (CFD) method coupled with K-K equation and the tortuous capillary pore diffusion model (TCPDM) was proposed for the simulations. The urea clearance rate predicted by the use of the numerical model agrees well with the experimental data, which verifies the validity of our numerical model. The distributions of concentration, pressure, and velocity in the hollow fiber membrane module were obtained to analyze the mass transfer behaviors of bilirubin and bovine serum albumin (BSA), and the effects of tube-side flow rate, shell-side flow rate, and fiber tube length on the bilirubin or BSA clearance rate were studied. The results show that the solute transport mainly occurred in the near inlet regions in the hollow fiber membrane module. Increasing the tube-side flow rate and the fiber tube length can effectively enhance the solute clearance rate, while the shell-side flow rate has less influence on the BSA clearance. The clearance of macromolecule BSA is dominated by convective solute transport, while the clearance of small molecule bilirubin is significantly affected by both convective and diffusive solute transport.

## 1. Introduction

According to the World Health Organization (WHO), more than 2 billion people are infected by the hepatitis B virus (HBV) in the world today. So far, there are about 296 million cases of chronic HBV infections worldwide, as well as about 58 million cases of hepatitis C virus (HCV) infections, and 1.1 million people die from HBV and HCV infections every year [1]. At present, there are over 300,000 new cases of liver failure reported annually in China, with a mortality rate as high as 70% [2]. Although liver transplantation is an important treatment for liver failure, the shortage of liver donors, difficulties in transplantation, and other factors prevent liver transplantation from meeting the requirements for treatment of liver diseases. There are approximately 2 million patients dying of acute kidney injury every year. The mortality rate is 10–30% for acute kidney injury patients without complication [3]. By 2030, 14.5 million people will have end-stage kidney disease, yet only 5.4 million will receive kidney replacement therapy due to economic, social, and political factors [4]. Under these circumstances, the research on artificial liver or kidney instruments plays an important role in the treatment of hepatic and kidney diseases.

The artificial liver is an extracorporeal mechanical, physical, and biological device that drives the patient’s blood flow through an external apparatus to remove toxins. It serves the purpose of temporarily assisting the function of the liver and works as a substitute for the human liver until the patient’s own liver function is restored or liver transplantation is performed. Since the artificial liver was first introduced in 1956, it has gone through different stages of development, starting from its emergence in the 1950s to the non-bioartificial liver based on the blood purification technology in the 1970s, and to the bioartificial liver and hybrid artificial liver developed over the past 20 years [5,6,7,8,9]. China has made numerous breakthroughs in the artificial liver system in the past 30 years. For example, the team led by Lanjuan Li successfully developed Li’s non-bioartificial liver, Li’s bioartificial liver, and Li’s hybrid artificial liver [10]. Bilirubin is commonly considered as an important marker for liver malfunction, as high blood bilirubin concentration can lead to hepatic encephalopathy or coma. However, bilirubin hemotoxin usually exists as an albumin-bound form in human plasma, and hence, the main objective of the artificial liver system is to eliminate the albumin-bound bilirubin [11]. Various artificial liver systems have been developed to remove albumin-bound bilirubin hemotoxin. MARS (Molecular Absorbent Recirculation System) utilizes a technique called albumin dialysis to dialyze out bilirubin using a concentrated albumin solution as the dialysate solution [12]. Instead of using albumin dialysate, the Prometheus system uses the membrane that allows the toxin-bound albumin proteins in the blood to selectively permeate to remove bilirubin [9]. The MARS and Prometheus systems are typical nonbiological artificial livers, and thus they are cell-free liver support devices. Bioartificial liver systems usually contain a compartmentalized bioreactor which incorporates liver cells, and thus they can also perform the biological roles of the liver, such as regulation and synthesis. Many bioartificial liver systems have been developed, such as ELAD (Extracorporeal Liver Assist Devices) [13], Hepat Assist system [14], BLSS (Bioartificial Liver Support System) [7], and MELS (Modular Extracorporeal Liver Support) [15]. Although many artificial liver systems have been invented, there has yet to be any definitive clinical data that confirm that these and other artificial livers can improve survival rates [11]. Therefore, artificial liver systems are still in the early stages and need to be improved, and more fundamental studies, such as study on mass transfer, need to be conducted for the optimum design of an artificial liver.

**Figure 1:**
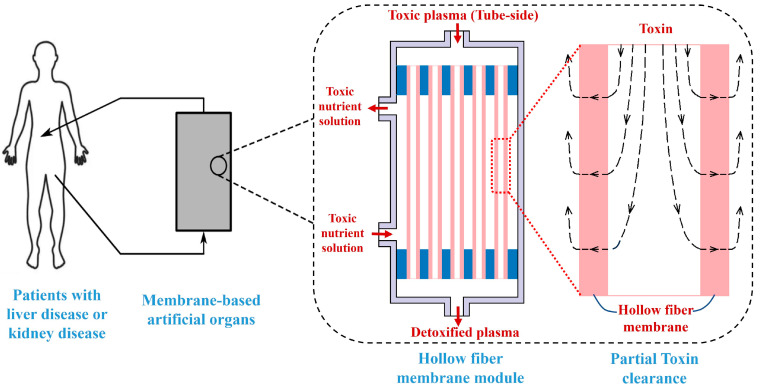
Schematic diagram of the hollow fiber membrane module for toxin clearance in artificial liver or artificial kidney system.

Significantly, most artificial liver devices, such as MARS, Prometheus, ELAD, Hepat Assist, and BLSS, are based on hollow fiber membrane technology. It also should be pointed out that hollow fiber membranes are commonly used in hemodialysis as artificial kidneys to treat renal disease, and in the late 1990s this technology was tentatively transferred to bioartificial applications for treating patients with liver disease, or other metabolic disorders [16,17,18]. For example, the hollow fiber dialysis membranes are used in both MARS and Prometheus artificial liver systems to remove toxins. The key difference of hollow fiber membranes between the artificial liver and the artificial kidney is the membrane pore size or molecular weight cut-off. The artificial kidney is usually used for removing small molecules such as urea and ammonia, and the molecular weight cut-off (MWCO) of dialyzer membranes for artificial kidney is from 3 kDa to around 65 kDa [19,20,21]. The artificial liver mainly needs to eliminate albumin-bound toxins such as bilirubin, and thus the MWCO of membranes for the artificial liver is usually larger than that for the artificial kidney, and its range is from 65 kDa to more than 1000 kDa—for example, MARS 65 kD [11], ELDA 70 kDa, BLSS 100 kDa [22,23], Prometheus 250 kDa [11], Hepat Assist ~5000 kDa [22]. The hollow fiber membrane module which is widely used to remove toxin for most artificial livers or artificial kidneys in clinical treatment is shown in Figure 1. The patient’s plasma flows through the tube-side region of the hollow fiber membrane module and enters the shell-side region through the membrane pores, and thus the toxins are removed from the patient’s body to achieve the goal of toxin clearance. Whether it is an artificial liver or an artificial kidney, a deep insight into the mass transfer characteristics in hollow fiber membrane module is very much needed for the design of hollow fiber-based devices.

A variety of numerical simulations and experimental research studies have been conducted to investigate the mass transfer behaviors in hollow fiber membrane modules of hollow fiber-based devices (such as artificial liver and artificial kidney). For hollow fiber membranes with intermediate molecular weight cut-off, most studies hold that convective solute transport plays a major role for mass transfer [24,25,26,27]. However, Macias et al. by employing a semi-empirical approach, found that intermediate molecular weight toxins are primarily removed by diffusive transport [28]. Legazpi et al. developed a mathematical model based on experimental data to assess the mass transfer efficiency of hollow fiber membranes, and the solute concentration distribution in the hollow fiber membrane module was in good agreement with experimental findings [29]. It should be pointed out that the mathematical model in Legazpi et al. only considered diffusive solute transport [29]. Sorrell et al. developed a mathematical model for the mass transfer behavior of an artificial liver hollow fiber membrane module, focusing on the transport of acetaminophen hepatotoxin and oxygen in the hollow fiber tubes, and their model assumed that the solute transport in the tube-side region is convective and diffusive, while only diffusive transport occurs in the regions of both hollow fiber membranes and shell-side [30]. In fact, convective transport also occurs in the regions of the shell-side and in the hollow fiber membrane. Ding et al. proposed a new numerical model for mass transfer in a hollow fiber membrane module, and the model consisted of two interpenetrating porous regions (i.e., blood flow region and dialysate flow region) that simulate the permeation flux across the porous membrane [31,32]. Although the validity of Ding’s numerical model was verified by experiments, their method does not reflect the membrane’s ability to remove solutes since the geometric arrangement of hollow fiber tubes in the hollow fiber membrane module is a parallel arrangement rather than an interpenetrating arrangement. Menshutina et al. used Ansys Fluent software 2020 R1 to simulate the flow of nutrient solution in a hollow fiber membrane module and obtained the optimal flow rate of the nutrient solution [33]. Sangeetha et al. used the finite software COMSOL Version 5.5 to analyze the blood flow in the tube-side region and the dialysate flow in the shell-side region, and found that the bent tubes had better toxin removal performance than the straight tube [34]. Gautier et al. established mathematical models to study the transport of vitamin B12 in three kinds of fiber membranes, i.e., microfiltration membrane, ultrafiltration membrane, and plasma separation membrane, and they found that convective transport is dominated in microfiltration membranes, and diffusive transport is dominated in ultrafiltration membranes, while both convective and diffusive transport occur in plasma separation membranes. They also pointed out that the mass transfer is mainly influenced by membrane type and operating conditions [18]. To study urea transport in the hollow fiber membrane, Magalhaes et al. proposed a new CFD model in which blood was treated as a multiphase fluid and the hollow fiber membrane was considered as an additional viscous resistance, and they obtained a new method for calculating clearance rate by changing the viscous resistance [35]. The numerical results in Magalhaes et al. were in agreement with experimental results of Liao et al. [36], but the diffusive solute transport was not included in the numerical model.

From the aforementioned research results, it can be seen that the mass transfer behavior in the hollow fiber membrane module of membrane-based artificial livers or artificial kidneys are still not very clear. At present, researchers mainly use experimental methods and empirical or semi-empirical mathematical models to obtain semi-empirical conclusions. Due to the complexity of the mass transfer, the transport behaviors in the hollow fiber membrane module still need to be revealed. Moreover, most of the studies did not take into account the curvature of the membrane pore. In fact, membrane pores are usually not completely straight, and the curvature of the membrane pore has a great impact on solute clearance. In the current work, in order to take into account the curvature of the membrane pores, a new computational fluid dynamics (CFD) method coupled with the tortuous capillary pore diffusion model (TCPDM) and K-K equation was proposed to simulate the mass transfer behaviors of macromolecule bovine serum albumin (BSA) and small molecule bilirubin in the hollow fiber membrane module. Although bilirubin exists as an albumin-bound form in blood plasma, it is strictly in thermodynamic equilibrium between the free bilirubin form and the albumin-bound bilirubin form, indicating that there is still a small amount of free bilirubin in the blood [11]. Hence, bilirubin and BSA were used to represent small and large molecules, respectively, to study the solute mass transfer behaviors in the hollow fiber membrane module.

This article is organized as follows. First, in Section 2, we l describe the numerical mode, and the validity of our numerical model is verified. Then, in Section 3, we present concentration distribution, pressure distribution, and velocity vector distribution in the hollow fiber membrane module to analyze the mass transfer behavior. The effects of tube-side flow rate, shell-side flow rate, and fiber tube length on mass transfer behavior are also discussed. Finally, the results are summarized in Section 4.

## 2. Numerical Method

### 2.1. Geometric Model

**Figure 2:**
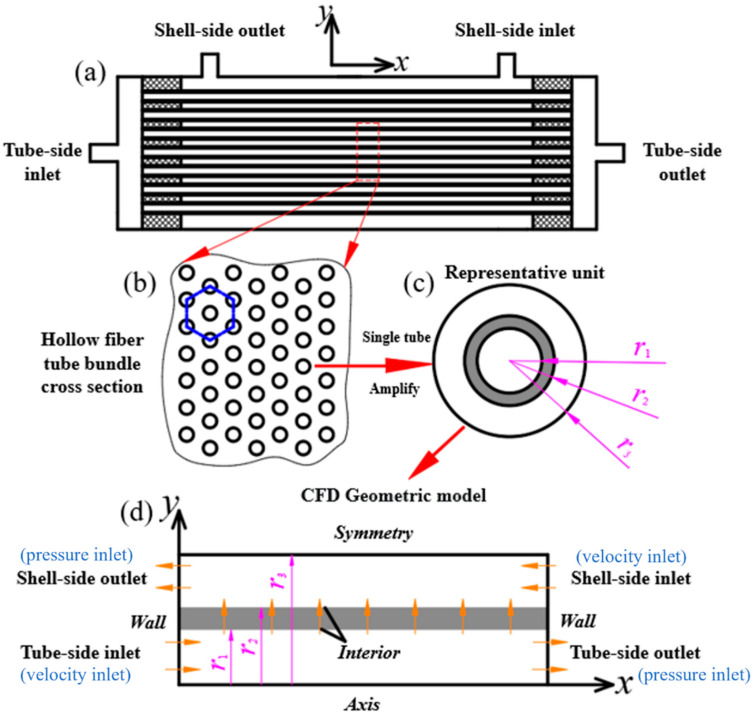
CFD geometric model for mass transfer study in hollow fiber membrane module (**a**) Hollow fiber membrane module; (**b**) hollow fiber tube bumdle cross section; (**c**) single tube; (**d**) two-dimensional axisymmetric model.

The schematic diagram of the hollow fiber membrane module is shown in Figure 2a. If the manufacturing tolerance is ignored, the hollow fiber tubes are typically uniformly arranged in the hollow fiber membrane module. Assuming that all hollow fiber tubes have the same diameter, the hollow fiber tubes can be considered as a triangular or hexagonal arrangement, as shown in Figure 2b. Due to this uniform arrangement, the hollow fiber tube bundle can be depicted by a representative unit, which includes the tube-side region, the membrane region, and the shell-side region, while the radii of these three regions are the inner radius of the hollow fiber tube $r_{1}$, the outer radius of the hollow fiber tube $r_{2}$, and the shell-side radius of the representative unit $r_{3}$, respectively, as shown in Figure 2c. The values of these three radii were set by using the literature-reported values of hollow fiber membrane module parameters [37]. Based on this representative unit, a CFD geometric model for mass transfer analysis of the hollow fiber membrane module can be constructed. Since the representative unit is axially symmetric, it can be further simplified into a two-dimensional axisymmetric model, as shown in Figure 2d. In the tube-side region, there is the flow of BSA solution or bilirubin solution, while the flow of nutrient solution (in this case, pure water) occurs in the shell side region. The flow directions of these two flows are opposite to each other. The membrane region serves as an interface for information exchange between the tube-side and shell-side regions, and the mesh in the membrane region is densified. The boundary conditions are shown in Figure 2d. The velocity inlet boundary condition is applied to the tube-side or shell-side inlet, and the pressure outlet boundary condition is applied to the tube-side or shell-side outlet. The interior boundary condition is applied to the interfaces of the membrane. A two-dimensional axisymmetric model is used and thus, there is an axis boundary condition. The symmetry boundary condition is used for the boundary between two adjacent representative units, and the wall boundary is used for both ends of the fiber membrane. Solute, such as bilirubin or BSA molecules, passes through membrane pores from the tube-side region to the shell-side region by convection and diffusion to achieve solute clearance.

### 2.2. Governing Equations

The continuity equation for the tube-side or shell-side region is as follows [31]:

(1) $$\frac{\partial v_{x}}{\partial x}+\frac{\partial v_{y}}{\partial y}=\frac{S_{v}}{\varepsilon }$$

where $v_{x}$ or $v_{y}$ is the velocity vector (*x* or *y* direction), $S_{v}$ represents the flux source term generated by the transmembrane fluid, and ε is the porosity factor of the membrane.

The momentum conservation equations for the tube-side and shell-side are as follows [38]:

(2) $$v_{x}\frac{\partial v_{x}}{\partial x}+v_{y}\frac{\partial v_{x}}{\partial y}=-\frac{1}{\rho }\frac{\partial p}{\partial x}+\frac{1}{\rho }\left(\frac{\partial \tau_{11}}{\partial x}+\frac{\partial \tau_{21}}{\partial y}\right)+S_{i}$$

(3) $$v_{x}\frac{\partial v_{y}}{\partial x}+v_{y}\frac{\partial v_{y}}{\partial y}=-\frac{1}{\rho }\frac{\partial p}{\partial y}+\frac{1}{\rho }\left(\frac{\partial \tau_{12}}{\partial x}+\frac{\partial \tau_{22}}{\partial y}\right)+S_{i}$$

where *p* is the pressure, τ is the shear stress, ρ is the density, $S_{i}$ represents the drag source term generated by the hollow fiber membrane.

The solute concentration in the tube-side and shell-side regions can be controlled by the following equation [38]:

(4) $$v_{x}\frac{\partial C_{b}}{\partial x}+v_{y}\frac{\partial C_{b}}{\partial y}=D_{s}\left(\frac{\partial^{2}C_{b}}{\partial x^{2}}+\frac{\partial^{2}C_{b}}{\partial y^{2}}\right)+\frac{S_{r}}{\varepsilon }$$

where $C_{b}$ is the solute concentration, $D_{s}$ is the solute diffusion coefficient, $S_{r}$ is the solute source term generated on the membrane. The solute source term $S_{r}$ has the following form:

(5) $$S_{r}=\frac{J_{s}}{∆\delta }$$

where $J_{s}$ is the solute transmembrane flux and ∆δ is the thickness of the hollow fiber membrane.

The K-K equation was developed by Kedem and Katchalsky to describe the mass transfer behavior in porous membranes [39]. The K-K equation is set up on the basis of continuum fluids theory. The diameter of the fiber membrane pore used in the current work is 39.5 nm, and the flow is continuous with this pore size. For the traditional K-K equation, the mass transport resistance for both the tube-side and shell-side regions is ignored. Thus, the total mass transfer coefficient is used to replace the solute permeability to modify the K-K equation, and the expressions are as follows [40]:

(6) $$J_{v}=L_{p}\times ∆p-\sigma RT\times ∆C$$

and

(7) $$J_{s}=\left(1-\sigma \right)C_{m}J_{v}+K_{0}\times ∆C$$

where $J_{v}$ is the convective transmembrane flux, $L_{p}$ is the hydraulic permeability of the porous membrane, ∆p is the transmembrane pressure (TMP), ∆C is the concentration difference between the two sides of the membrane, σ is the reflection coefficient of the solute, R is the gas constant, *T* is the absolute temperature, $C_{m}$ is the average solute concentration in the porous membrane, and $K_{0}$ is the total mass transfer coefficient of solute diffusion through the porous membrane. Among the above parameters, the hydraulic permeability $L_{p}$ is the intrinsic parameter of the membrane, and the reflection coefficient of the solute is related to the total mass transfer coefficient, the membrane pore size, and the solute molecule size. The work of Anderson describes the relationship between the pore size and the solute molecule size, and this relationship is expressed as follows [41,42]:

(8) $$\sigma ={\left(1-{\left(1-\frac{r_{s}}{r_{p}}\right)}^{2}\right)}^{2}$$

where $r_{p}$ is the radius of the membrane pore, and $r_{s}$ is the Stokes radius of the solute molecule. For small solute molecule size, the value of σ is very small and can be ignored when the membrane pore radius is about 20 nm or larger [38]. Therefore, the above Equation (6) can be further simplified as follows:

(9) $$J_{v}\;\approx \;L_{p}\times ∆p$$

The pipeline type membrane pores are usually used to model membrane pores in numerical simulation. Thus, in order to take into account the curvature of the hollow fiber membrane pores, the tortuous capillary pore diffusion model (TCPDM) was coupled in our numerical model. The calculation formulas of the hydraulic permeability coefficient $L_{p}$ and the diffusion permeability $K_{0}$ in TCPDM are expressed as below [43]:

(10) $$L_{p}=\frac{r_{p}^{2}\varepsilon }{8\psi \mu ∆\delta }$$

(11) $$K_{0}=\frac{D_{0}}{∆\delta }$$

(12) $$D_{0}=\frac{D_{s}F\left(q\right)S_{D}}{\psi }$$

(13) $$F\left(q\right)=\frac{1-2.1050q+2.0865q^{3}-1.7068q^{5}+0.72603q^{6}}{1-0.75857q^{5}}$$

(14) $$S_{D}={\left(1-q\right)}^{2}$$

(15) $$q=\frac{r_{s}}{r_{p}}$$

where $D_{0}$ is the effective diffusivity, $D_{s}$ is the volume diffusivity, $F\left(q\right)$ is the friction coefficient, $S_{D}$ is the steric hindrance factor at the pore entrance for the diffusive transport, and *ψ* is the tortuous factor of the membrane pores. The effective diffusivity $D_{0}$ is obviously smaller than the volume diffusivity $D_{s}$. The friction coefficient $F\left(q\right)$ is used to describe the friction between the solute molecule and the membrane pore wall. As can be seen from Equations (10) and (12), both the hydraulic permeability coefficient $L_{p}$ and the diffusion permeability $K_{0}$ can be reduced when the tortuous factor of the membrane pore is introduced, and thus the curvature of the membrane pore will have a great impact on solute clearance. In fact, membrane pores are usually not completely straight. When ignoring the curvature of the membrane pore, the simulated solute clearance will be higher than the actual clearance.

For different solutes, the diffusion coefficients are different, and the diffusion coefficient is very important for the simulation results. The expression of the diffusion coefficient of the biomolecule used in the simulation is as follows [44]:

(16) $$D=1.62\times 10^{-4}{\left(MW\right)}^{-0.552}$$

where *MW* is the molecular weight of solute. The molecular weight and the Stokes radius of bilirubin and BSA are shown in Table 1.

The hollow fiber membrane is a porous medium. In CFD simulations, the porous medium model is usually used to simulate the porous region by adding a drag source term (see $S_{i}$ in Equations (2) and (3)) to the momentum equation. This amounts to introducing an additional flow resistance to the CFD model. The drag source term $S_{i}$ consists of two parts, namely, the viscous loss term (Darcy) and the inertial loss term, expressed by the following:

(17) $$S_{i}=-\left(\sum_{j=1}^{2}D_{ij}\times \mu \times v_{j}+\sum_{j=1}^{2}C_{ij}\frac{1}{2}\rho \times \left|v\right|\times v_{j}\right)$$

where $S_{i}$ is the drag source term for the i (*x* or *y* direction) momentum equations, $\left|v\right|$ is the absolute value of the velocity, and μ is the viscosity.

For the homogeneous porous medium, the above equation can be simplified as follows:

(18) $$S_{i}=-\left(\frac{\mu }{\alpha }\times v_{i}+C_{2}\frac{1}{2}\left|v\right|\times v_{i}\right)$$

where α is the permeability of the porous medium, 1/α is the viscous drag coefficient, and $C_{2}$ is the inertial drag coefficient. In the anisotropic porous medium, if the drag coefficient in one direction is much larger than that in other directions, it is not necessary to set a large value for the drag coefficient. The drag coefficient can be set to 2–3 orders of magnitude of that in other directions. For laminar flow, the constant $C_{2}$ can be ignored and the pressure drop is proportional to the velocity. Ignoring the convective term and diffusion term, Darcy’s law can be used to describe the flow in porous medium, expressed by the following:

(19) $$\nabla p=-\frac{\mu }{\alpha }\vec{v}$$

Therefore, the pressure drop of porous medium can be given by the following formulas:

(20) $$\nabla p_{x}=\sum_{j=1}^{3}\frac{\mu }{\alpha_{xj}}\vec{v_{j}}∆\delta_{x}$$

(21) $$\nabla p_{y}=\sum_{j=1}^{3}\frac{\mu }{\alpha_{yj}}\vec{v_{j}}∆\delta_{y}$$

where $∆\delta_{x}$ and $∆\delta_{y}$ are the thickness of the porous medium in *x* and *y* directions, respectively.

The clearance rate of the hollow fiber membrane module can be determined by calculating the average concentration of solutes at the inlet and outlet of the tube-side. The calculation formula of the clearance rate of the hollow fiber membrane module is as below [35]:

(22) $${CL}_{S}=\frac{Q_{B,in}{\bar{C}}_{s,in}-Q_{B,out}\;{\bar{C}}_{s,out}}{{\bar{C}}_{s,in}}$$

where $Q_{B,in}$ is the flow rate at the tube-side inlet, $Q_{B,out}$ is the flow rate at the tube-side outlet, ${\bar{C}}_{s,in}$ is the average concentration of solute at the tube-side inlet, and ${\bar{C}}_{s,out}$ is the average concentration of solute at the tube-side outlet.

### 2.3. Model Parameters and Mesh Independence Verification

The ANSYS-FLUENT software package was used to solve the above numerical model in this study, and the user-defined function (UDF) was employed to modify the diffusive mass transfer of solutes across the hollow fiber membrane. Various model parameters used in the simulations are shown in Table 2, and some of the parameters were obtained according to Islam et al. [37] and Yamamoto et al. [43]. The normal concentration of bilirubin in the human body ranges from 3.7 to 17.1 μmol/L. Considering an increase in bilirubin concentration in patient plasma, a concentration of $1\times 10^{-4}$ mol/L was used in the simulation for this study. Since BSA is similar to human blood albumin and the albumin concentration in human plasma ranges from 35 to 51 g/L, a BSA concentration of 0.000597 mol/L was employed in this study.

**Figure 3:**
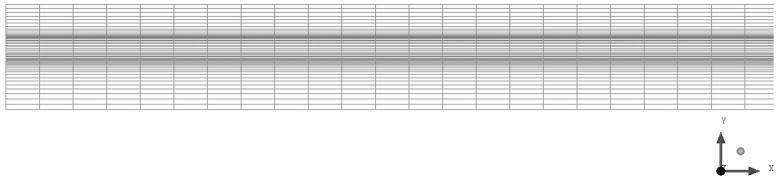
Schematic diagram of mesh for the hollow fiber membrane module.

The two-dimensional axisymmetric model of the hollow fiber membrane module established in this study was meshed by ICEM software, and quadrilateral mesh was used for meshing. Figure 3 shows the schematic diagram of the two-dimensional axisymmetric mesh for the hollow fiber membrane module (only a section of the model is displayed in Figure 3). Because of the complexity of the flow in the membrane region, the mesh in the membrane region is densified to ensure the accuracy of the simulation. As can be seen in Figure 3, the grid is densified near the membrane region.

**Figure 4:**
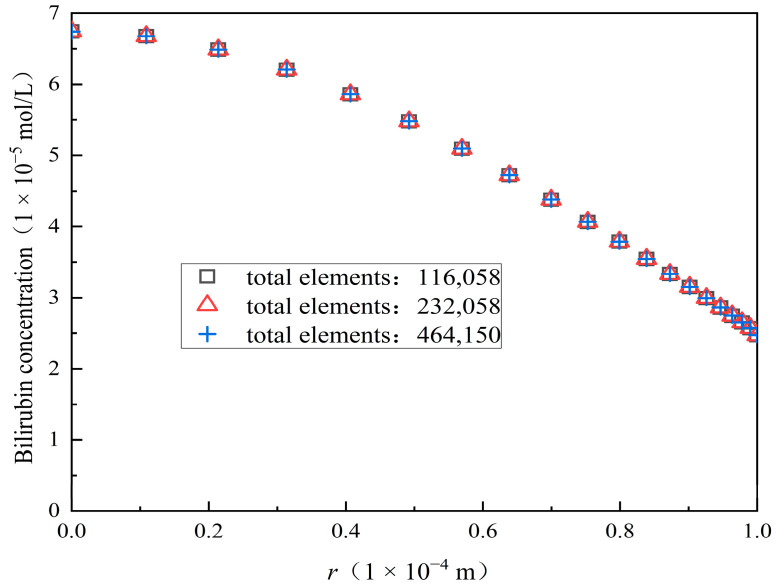
The bilirubin concentration distributions at the tube-side outlet for three different total mesh elements.

For mesh independence verification, three different numbers of total mesh elements, i.e., 116,058, 232,058, and 464,150, are used for the simulations. Figure 4 shows the bilirubin concentration distributions along the radial direction for the three different total mesh elements and Table 3 gives the maximum concentration at the tube-side outlet and relative errors between two different total mesh elements. As we can see from Figure 4 and Table 3, the differences of bilirubin concentration among the three different total mesh elements are very small, and the maximum concentration tends to a constant value with the increase of mesh elements. The relative error between the calculation results of 116,058 and 232,058 is 0.008007%, and the relative error between the calculation results of 232,058 and 464,150 is 0.004152%. It can be seen that the simulation results are independent of these three different total mesh elements. In order to reduce the computational time, we used 232,058 total mesh elements for the simulation of the mass transfer behavior in the hollow fiber membrane module.

### 2.4. Validity of Numerical Model

**Figure 5:**
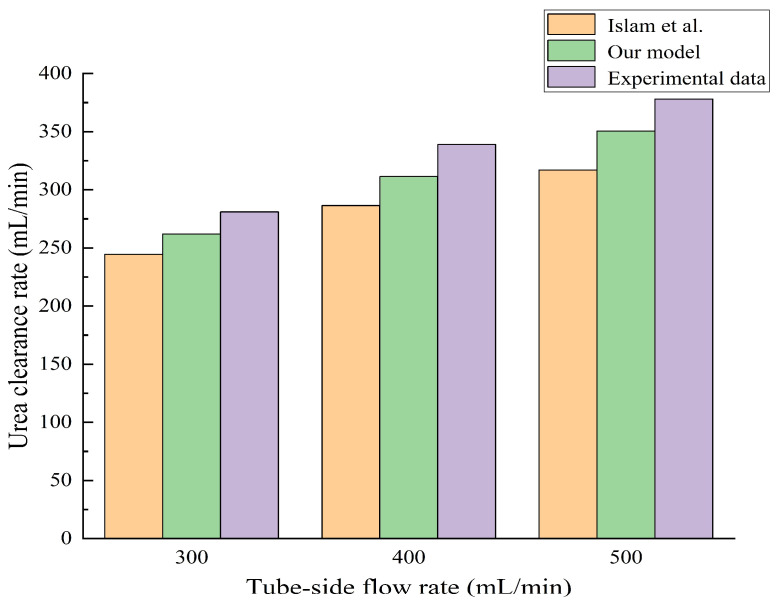
The urea clearance rates obtained by Islam et al. [37], our model, and experimental observation by manufacturer [47], respectively.

To verify the validity of the proposed numerical model, the urea clearance rate predicted by our numerical model was compared with the numerical simulation results obtained by Islam et al. [37] and the experimental results provided by the manufacturer [47], as shown in Figure 5. Three cases with different working conditions (i.e., with the tube-side flow rates 300, 400, and 500 mL/min) were selected for data comparison. The simulation parameters are shown in Table 2. The molecular weight and the Stokes radius $r_{s}$ of the urea molecule are 60 Da and 0.24 nm, respectively. Table 4 shows the specific clearance rates and the relative errors for our model and the literature data. As can be seen in Figure 5 and Table 4, the urea clearance rate predicted by our numerical model was in agreement with the experimental result. For the tube-side flow rate with 300 mL/min, the urea clearance rates obtained, by Islam et al. [37], by our model, and by experimental observation are 244.62 mL/min, 261.99 mL/min, and 281 mL/min, respectively. The relative error between the simulation result by Islam et al. [37] and the experimental data is 12.95%, while it is only 6.77% between the simulation result of our model and the experimental data. This indicates that our numerical model is better than the numerical model proposed by Islam et al. [37]. Hence, the validity of our numerical model was verified. It should be pointed out that a theoretical model is difficult to map perfectly to a real physical system. That is to say, the tortuous capillary pore diffusion model (TCPDM) may not perfectly match with the shapes of the actual fiber membrane pores, which would lead to the relative error. Moreover, the actual pore size distribution in the hollow fiber membrane is not uniform, with some pores having large pore size and some pores having small pore size. In the current work, all the pore sizes are assumed to be the same for simplicity in the simulations. This could be another reason for the cause of the relative error.

## 3. Results and Discussion

### 3.1. Mass Transfer Behaviors of Bilirubin

**Figure 6:**
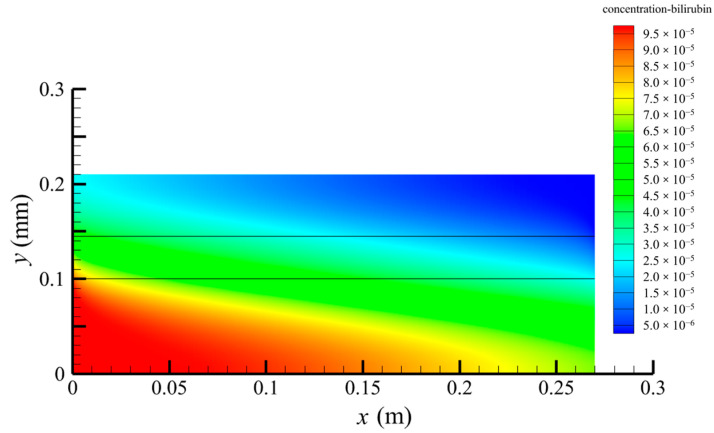
The bilirubin concentration distribution in the hollow fiber membrane module.

Figure 6 shows the bilirubin concentration distribution in the hollow fiber membrane module. As seen in Figure 6, the bilirubin enters at the tube-side inlet, but the bilirubin was found both in the membrane region and in the shell-side region. It means that bilirubin transport from the tube-side to the shell-side has occurred. The bilirubin concentration decreases with increasing axial or radial distance, and the decrease in the radial direction is much faster than that in the axial direction. This is due to the fact that the membrane acts as a barrier to the solute, which can reduce the bilirubin transport through the membrane. From the bilirubin concentration distribution shown in Figure 6, it can also be observed that the bilirubin transport from the tube-side to the shell-side has mainly occurred in the regions near the tube-side inlet, indicating that the removal of bilirubin primarily takes place near the tube-side inlet.

**Figure 7:**
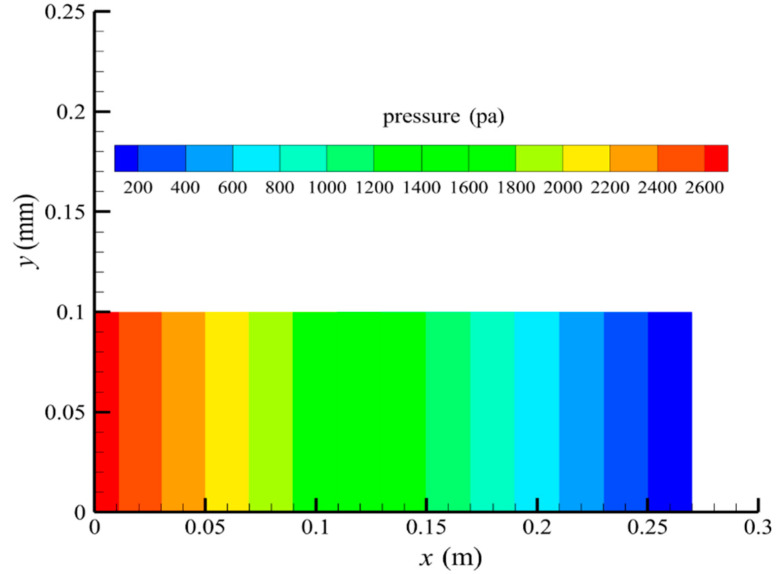
The pressure distribution in the tube-side region.

**Figure 8:**
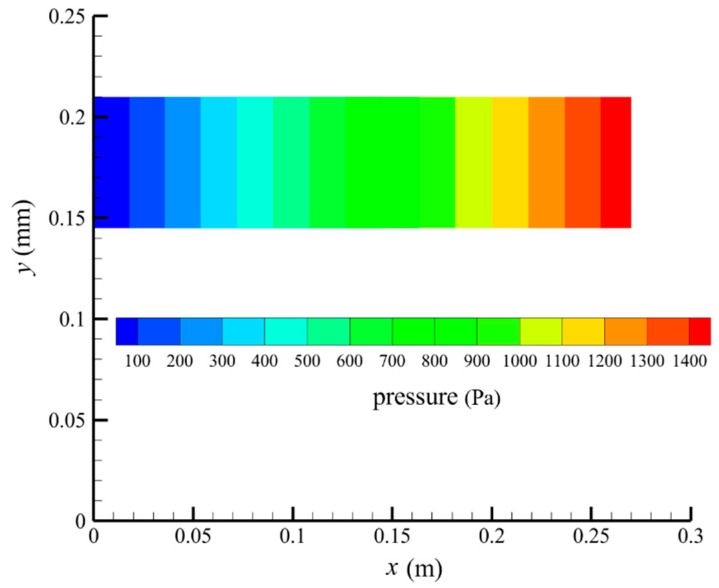
The pressure distribution in the shell-side region.

Figure 7 shows the pressure distribution in the tube-side region, and Figure 8 presents the pressure distribution in the shell-side region. As can be seen in Figure 7 and Figure 8, the pressure is highest at the inlet of both the tube-side and the shell-side, and decreases along the axial direction. However, the pressure remains almost constant in the radial direction. This is because the pore sizes of the hollow fiber membrane are small, resulting in a low flow rate in the hollow fiber membrane pores. As a result, the pressure drop from the axis to the membrane surface is small, and hence the pressure along the radial direction in the hollow fiber membrane module remains nearly constant. Therefore, the transmembrane pressure (TMP) between the tube-side and the shell-side can be easily estimated. TMP can drive the solute to penetrate into the shell-side region through the membrane pores. The calculation of TMP can be expressed by the following:

(23) $$∆p\approx p_{axis}-p_{symmetry}$$

where $p_{axis}$ is the pressure at the axis of the geometric model, and $p_{symmetry}$ is the pressure at the symmetry of the geometric model (see Figure 2). If ∆p>0, TMP is defined as positive TMP, and if ∆p<0, TMP is defined as negative TMP, then positive TMP conducts the convective mass transfer from the tube-side to the shell-side, and negative TMP conducts the convective mass transfer from the shell-side to the tube-side.

**Figure 9:**
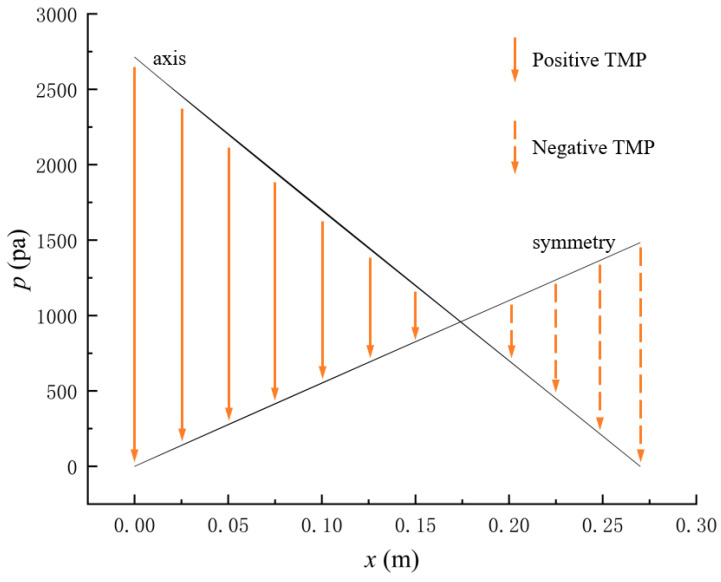
The pressure distributions along the axis and the symmetry.

Figure 9 displays the pressure distribution along the axis and the symmetry, and the left side of the intersection of the two pressure profiles is the positive TMP region and the right side is the negative TMP region. It can be seen that the pressure distribution along the axial and the symmetry is almost linear. Hence, according to Equation (23), TMP distribution is also almost linear. Consequently, the convective transmembrane flux exhibits a linear distribution, as described in Equation (9). This result is consistent with the findings of Magalhaes et al. [35]. As shown in Figure 9, at *x ≈* 0~0.175 m, the pressure in the tube-side region is higher than that in the shell-side region. The positive TMP drives the bilirubin to transfer from the tube-side region through the fiber membrane to the shell-side region. Due to this positive TMP, the bilirubin convective transport has mainly occurred in the regions near the tube-side inlet, which is consistent with the bilirubin concentration distribution shown in Figure 6. At *x ≈* 0.175~0.27 m near the inlet of the shell-side region, the negative TMP drives the fluid or solute to transfer from the shell-side region through the fiber membrane to the tube-side region. Therefore, at *x ≈* 0.175~0.27 m, the bilirubin molecule cannot transfer from the tube-side region to the shell-side region via convective mass transfer. However, as can be observed in Figure 6, bilirubin molecules still exist in the membrane region and the shell-side region at *x ≈* 0.175~0.27 m, indicating that the bilirubin can also be transferred from the tube-side region to the shell-side region via diffusive mass transfer.

**Figure 10:**
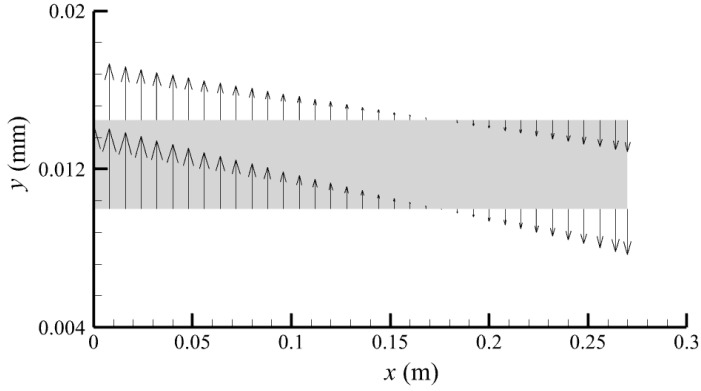
The velocity vector distribution at the interfaces of fiber membrane.

Figure 10 displays the velocity vector distribution at the interfaces of the fiber membrane. As can be seen in Figure 10, it is obvious that mass transfer has mainly occurred in the regions near the tube-side or shell-side inlet. This is attributed to the larger TMP near the tube-side or shell-side inlet. The velocity vector distribution is consistent with the bilirubin concentration distribution shown in Figure 6 and the pressure distribution shown in Figure 9.

### 3.2. Effect of Tube-Side Flow Rate on Mass Transfer

**Figure 11:**
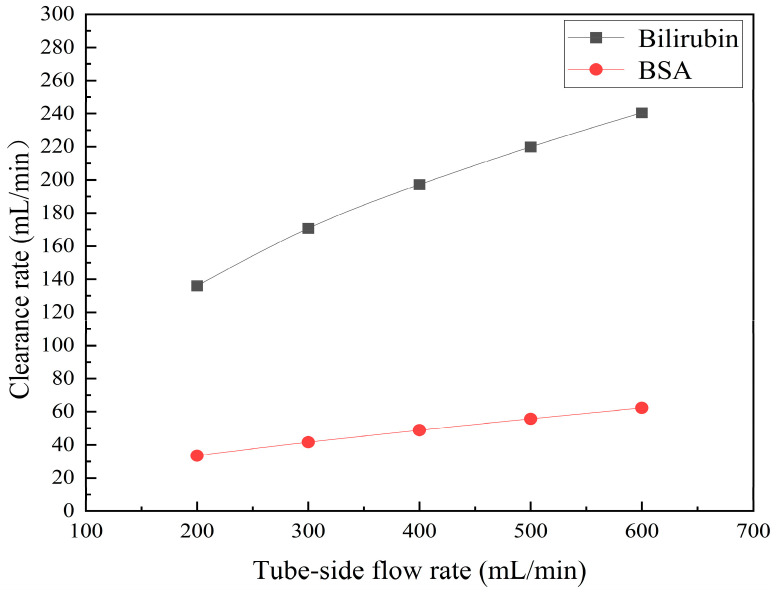
The bilirubin and BSA clearance rates for varying tube-side flow rates: 200, 300, 400, 500, and 600 mL/min..

**Figure 12:**
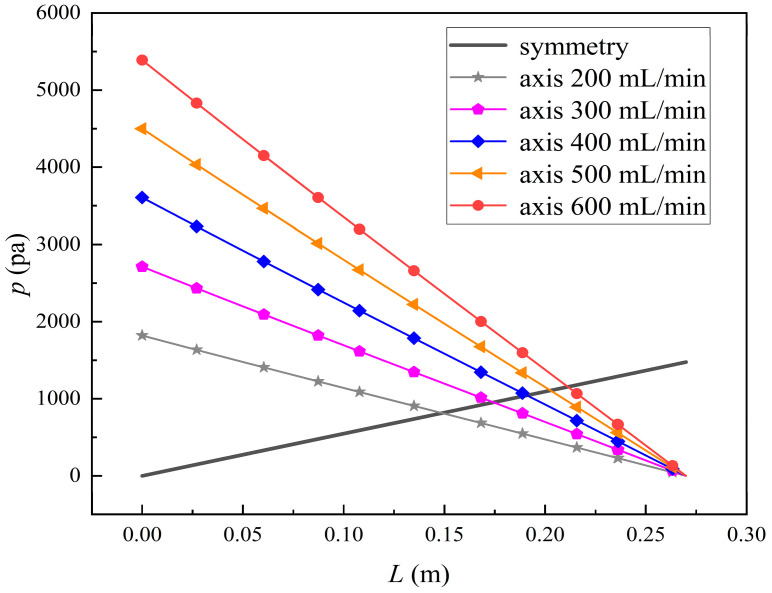
The pressure distributions along the axis and the symmetry for varying tube-side flow rates.

Figure 11 shows the bilirubin and BSA clearance rates for varying tube-side flow rates (i.e., 200, 300, 400, 500, and 600 mL/min) with the shell-side flow rate 500 mL/min. Table 5 shows the bilirubin and BSA concentration distributions for varying tube-side flow rates. As can be seen in Table 5, with the increase of the tube-side flow rate, the bilirubin or BSA concentration increases in the tube-side region and at the tube-side outlet, but the concentration also increases in the membrane region and the shell-side region. As can also be seen in Figure 11, both the bilirubin and the BSA clearance rate increase with the increase of the tube-side flow rate. Specifically, the bilirubin clearance rate increases from 136.05 to 240.54 mL/min, while the BSA clearance rate increases from 33.54 to 62.30 mL/min. This is because both the diffusive and convective transport increase with the increase in the tube-side flow rate. The concentration gradient between the tube-side region and the shell-side region increases with the increase in the tube-side flow rate, and hence the solute diffusion flux increases. At the same time, the positive TMP also increases from 1476.48 to 5389.02 Ρa with the increase in the tube-side flow rate, as shown in Figure 12, and hence, the solute convective flux increases. Furthermore, Figure 12 also shows that the abscissa value of the intersection of the two pressure distribution profiles increases with the increase of the tube-side flow rate. This means that the positive TMP region increases, namely, the convective flux area increases. Due to the increase of the tube-side flow rate, the cleared bilirubin is quickly supplemented, leading to the increase of the concentration gradient between the tube-side and shell-side, and thus the solute diffusion flux has been increased. Therefore, the clearance rate of bilirubin and BSA increased with the increase of tube-side flow.

Figure 11 also shows that the clearance rate of bilirubin molecules (low molecular weight) exhibits faster increase than that of BSA molecules (high molecular weight) with the increase in tube-side flow rate, while the bilirubin clearance rate is much higher than the BSA clearance rate. For a BSA molecule, due to its large molecular size, the values of the friction coefficient F(q) and steric hindrance factor $S_{D}$ are small according to Equations (11)–(15). This implies that the diffusion flux of BSA becomes smaller, and hence, the increase in clearance rate of BSA solute is not as significant as that of bilirubin. Therefore, it can be concluded the BSA transport is dominated by convective mass transfer. In order to increase the clearance rate of BSA, it is necessary to enhance the convective transport, by increasing the pore diameter of the fiber membrane.

### 3.3. Effect of Shell-Side Flow Rate on Mass Transfer

**Figure 13:**
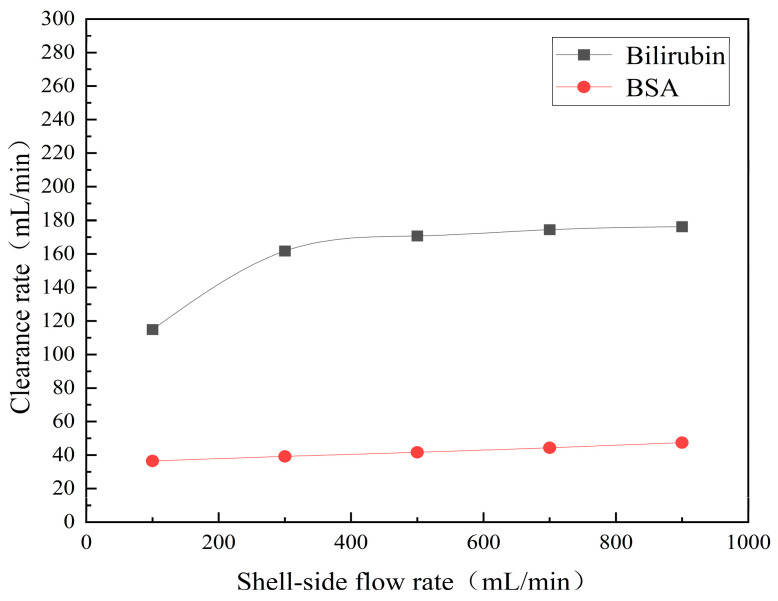
The bilirubin and BSA clearance rates for varying shell-side flow rates: 100, 300, 500, 700, and 900 mL/min..

Figure 13 shows the bilirubin and BSA clearance rates for varying shell-side flow rates (i.e., 100, 300, 500, 700, and 900 mL/min) with the tube-side flow rate 300 mL/min. Table 6 shows the bilirubin and BSA concentration distributions for varying shell-side flow rates. It can be observed in Table 6 that the concentration at the tube-side outlet and in the tube-side region decreases with the increase in the shell-side flow rate, indicating that the clearance efficiency of the solute improves. As shown in Figure 13, with the increase in the shell-side flow rate, the bilirubin clearance rate exhibits a rapid increase from 114.93 to 170.79 mL/min when the shell-side flow rate is less than 300 mL/min and then a slow increase from 170.79 to 176.34 mL/min, while the BSA clearance rate increases slowly from 36.46 to 47.46 mL/min.

**Figure 14:**
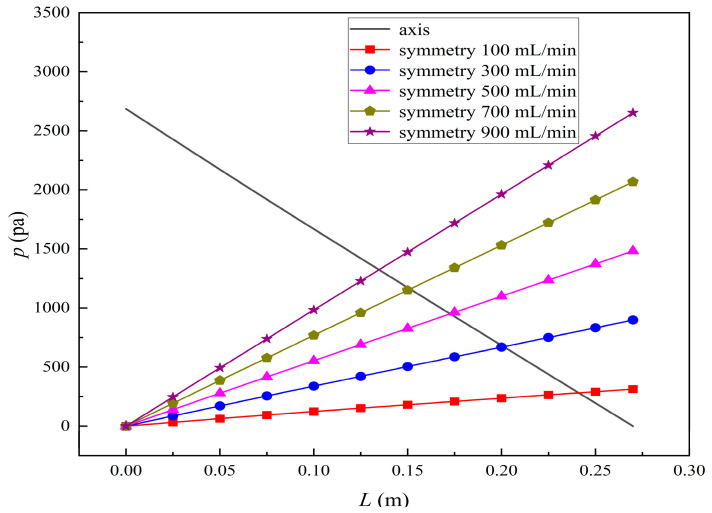
The pressure distributions along the axis and the symmetry for varying shell-side flow rates.

With the increase in shell-side flow rate, the solute molecules in the shell-side region are quickly taken away, leading to a decrease in solute concentration both in the shell-side and fiber membrane regions, as shown in Table 6. This would lead to the increase of the concentration gradient between the tube-side region and the shell-side region. Due to the increase of the concentration gradient, the clearance rate can be increased by diffusive transport. However, with the increase in the shell-side flow rate, the positive TMP decreases, and the positive TMP region also decreases since the abscissa value of the intersection of the two pressure distribution profiles decreases, as shown in Figure 14. The decreases of both the positive TMP and the positive TMP region would result in the decrease of the solute convective flux. Therefore, a larger shell-side flow rate will lead to a smaller convective flux and a larger diffusive flux. The diffusion clearance of solute increases and the convection clearance decreases with the increase of shell flow rate. For small bilirubin, due to its large diffusion coefficient, the increase of the diffusion clearance is greater than the decrease of the convection clearance. Therefore, with the increase of shell-side rate, the clearance of bilirubin still increases. As can be seen from Figure 14, with the continuous increase of shell-side rate, the positive TMP decreases (this positive TMP leads to the convection clearance), and thus the convection clearance is becoming smaller and smaller. Meanwhile, the gradient of bilirubin concentration reaches an asymptotic value, which means the diffusion clearance rate tends towards a stable value. This is the reason why the bilirubin clearance rate exhibits a slow increase with a large shell-side flow rate, see Figure 13. Although the convective flux decreases with the increase in the shell-side flow rate, the bilirubin clearance rate still increases, especially for the shell-side flow rate of less than 300 mL/min. This is due to the increase in shell-side flow rate, resulting in an elevated concentration gradient, thus leading to greater diffusion; so diffusive transport also plays an important role for bilirubin mass transfer. For BSA, the clearance rate shows only a slight increase, indicating that the mass transfer of BSA is primarily driven by convection and slightly driven by diffusion, consistent with the conclusion of the previous subsection. A smaller convective flux caused by a larger shell-side flow rate leads to a slight increase in the BSA clearance rate. These results further demonstrate that the clearance of the macromolecule BSA is dominated by convective transport, while the clearance of the small molecule bilirubin is significantly affected by both convective and diffusive transport.

### 3.4. Effect of Fiber Tube Length on Mass Transfer

**Figure 15:**
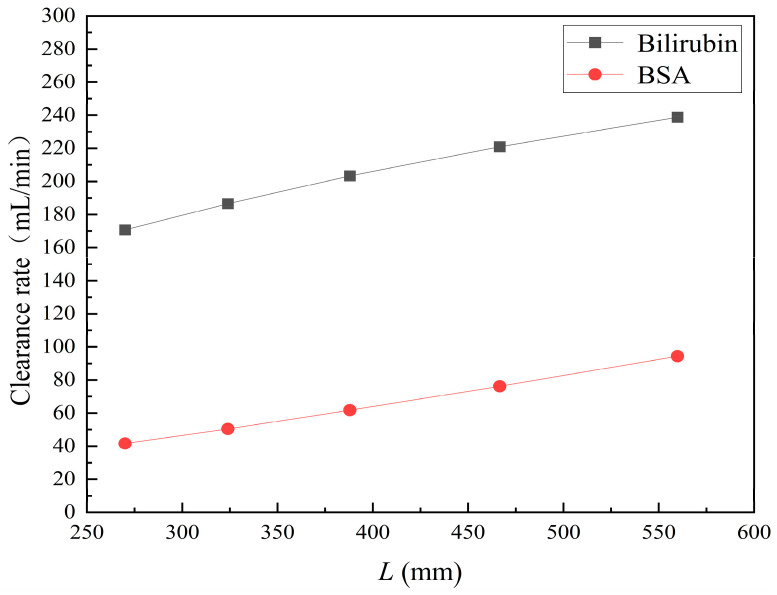
The bilirubin and BSA clearance rates for varying fiber tube lengths: 270, 324, 388.8, 466.56, and 559.872 mm.

Figure 15 shows the bilirubin and BSA clearance rates for varying fiber tube lengths (i.e., 270, 324, 388.8, 466.56, and 559.872 mm) with the tube-side flow rate 300 mL/min and the shell-side flow rate 500 mL/min. The fiber tube length is increasing proportionally with the ratio 1.2. Table 7 shows the bilirubin and BSA concentration distributions for varying fiber tube lengths. It can be seen from Figure 15 that with the increase in the fiber tube length, the bilirubin clearance rate increases from 170.79 to 238.786 mL/min, while the BSA clearance rate increases from 41.67 to 94.36 mL/min. Clearance rates of both bilirubin and BSA have been obviously increased, which is consistent with the bilirubin and BSA concentration distributions in Table 7.

**Figure 16:**
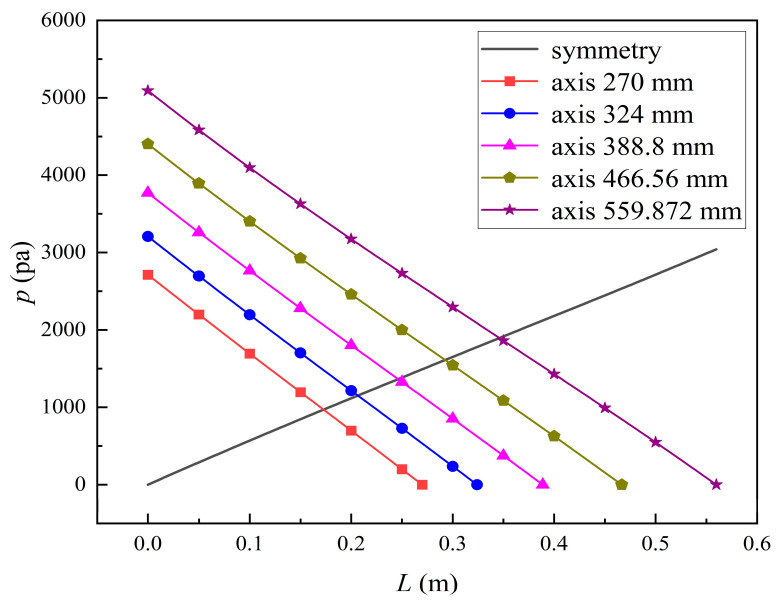
The pressure distributions along the axis and the symmetry for varying fiber tube lengths.

Increasing the fiber tube length would lead to an increase in the contact area between the solute and the membrane, namely, an increase in the diffusive and convective flux area. Hence, both diffusive and convective transport were strengthened due to the larger flux area. On the other hand, as the fiber tube length increases, the pressure drop in the axial direction also increases, which would lead to a larger positive TMP for enhancing the convective transport. Figure 16 shows the pressure distributions along the axis and the symmetry for varying fiber tube lengths. In Figure 16, the pressure profiles along the symmetry for varying fiber tube lengths are overlapped and thus, only one profile is displayed. As shown in Figure 16, both the positive TMP and the positive TMP region increase as the fiber tube length increases, and thus the bilirubin or BSA convective flux increases. The above two factors can result in a significant increase in in the diffusive and convective flux, and thus the bilirubin or BSA clearance rate is obviously increased as the fiber tube length increases.

It should also be pointed out that although increasing the fiber tube length can improve toxin clearance, a longer fiber tube requires a higher driving pressure to produce fluid flow, leading to higher equipment costs and energy consumption. Therefore, the fiber tube length design needs to strike a balance between toxin clearance and economic cost.

To summarize, Table 8 shows the bilirubin or BSA molecule characteristics, membrane pore size, and the corresponding mass transfer form. The removal of both small and large molecules can be achieved by increasing the tube-side flow rate or the fiber tube length. Increasing the shell-side flow rate can help to remove small molecules but it cannot help to remove large molecules.

## 4. Conclusions

In this work, we proposed a new CFD method to investigate the mass transfer behavior in a hollow fiber membrane module for membrane-based artificial organs. The K-K equation was used to describe the convective and diffusive mass transfer, and TCPDM was coupled to the CFD model to take into account the curvature of the membrane pores. The results of the bilirubin and BSA mass transfer behavior in the hollow fiber membrane module were obtained and the effects of tube-side flow rate, shell-side flow rate, and fiber tube length on the solute clearance rate were discussed. The following conclusions were obtained:

(1) The urea clearance rate obtained by our numerical model agrees well with the experimental data and the numerical simulation results. When the tube-side flow rate is 300 mL/min, 400 mL/min, and 500 mL/min, the relative errors between our simulation results and the experimental data are 6.77%, 8.13%, and 7.27%, respectively.
(2) The solute concentration in the hollow fiber membrane module decreases with increasing the axial or radial distance, and the decrease in the radial direction is much faster than that in the axial direction. The mass transfer mainly occurred in the regions near the tube-side or shell-side inlet of the hollow fiber membrane module.
(3) The clearance of macromolecule BSA is dominated by convective transport, while the clearance of small molecule bilirubin is significantly affected by both convective and diffusive transport. A larger tube-side flow rate or fiber tube length will lead to a larger convective flux and a larger diffusive flux, and thus the clearance of both bilirubin and BSA is improved with the increase in the tube-side flow rate or the fiber tube length. For the shell-side flow rate, a larger shell-side flow rate will lead to a smaller convective flux and a larger diffusive flux. On increasing the shell-side flow rate, the bilirubin clearance rate exhibits a rapid increase and then a slow increase, while the BSA clearance rate shows only a slight increase.

Our numerical simulation results can provide in-depth understanding of the mass transfer mechanisms and thus help to design optimally the hollow fiber membrane module and operating parameters for an artificial liver or artificial kidney. Although the proposed CFD model obtained good results, proposing a more accurate tortuous capillary pore diffusion model (TCPDM) to match perfectly with the curvature of the membrane pore would help provide a more accurate simulation result. Furthermore, membrane fouling was not considered in the current simulation work. In fact, biomolecules such as bilirubin and BSA may be adsorbed by the membrane surface, leading to membrane fouling and thus affecting the mass transfer of the fiber membrane. Therefore, a more accurate CFD model including membrane fouling needs to be developed in future research.

## Figures and Tables

**Table 1:** The molecular weight, the Stokes radius, and diffusion coefficient of bilirubin and BSA [45,46].

Solute	*MW* (Da)	$r_{s}$ (nm)	Diffusion Coefficient ($m^{2}/s$)
Bilirubin	584.66	0.5	$4.810\times 10^{-10}$
BSA	67,000	3.61	$3.5117\times 10^{-11}$

**Table 2:** Various parameters used in the simulations.

Parameters	Values	Units
Length of the fiber, *L*	270	Mm
Inner radius of the hollow fiber tube, $r_{1}$	0.1	Mm
Outer radius of the hollow fiber tube, $r_{2}$	0.145	Mm
Shell-side radius of the representative unit, $r_{3}$	0.210	Mm
Tube-side inlet flow rate, $Q_{B,in}$	300	mL/min
Shell-side inlet flow rate, $Q_{D,in}$	500	mL/min
Bilirubin concentration, $C_{in}$	1 $\times \;10^{-4}$	mol/L
BSA concentration, $C_{in}$	0.000597	mol/L
Urea concentration, $C_{in}$	1	mol/L
Total number of fiber tubes, N	12,000	
Porosity factor, ε	0.1	
Diameter of the fiber membrane pore, $d_{p}$	39.5	Nm
Tortuous factor, ψ	2.27	

**Table 3:** The maximum concentration at the tube-side outlet and relative error between two different total mesh elements.

Total Elements	Maximum Concentration (mol/L)	Relative Error
116,058	6.74401 × 10^−5^	
		0.008007%
232,058	6.74347 × 10^−5^	
		0.004152%
464,150	6.74319 × 10^−5^	

**Table 4:** Comparisons of our model results with literature data.

Tube-Side Flow Rate (mL/min)	Our Model Predicted (mL/min)	Islam et al. [37] (mL/min)	Experimental Data [47] (mL/min)	Relative Error between Our Model and Experimental Data	Relative Error between Islam et al. and Experimental Data
300	261.99	244.62	281	6.77%	12.95%
400	311.43	286.40	339	8.13%	15.52%
500	350.53	317.04	378	7.27%	16.13%

**Table 5:** The bilirubin and BSA concentration distributions for varying tube-side flow rates.

Tube-Side Flow Rate mL/min	Bilirubin	BSA
200	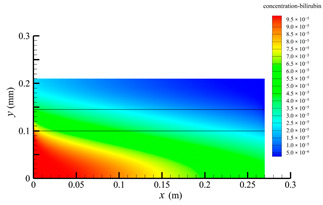	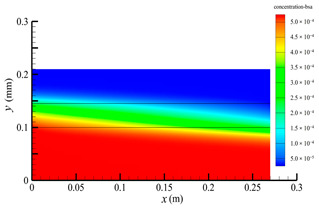
300	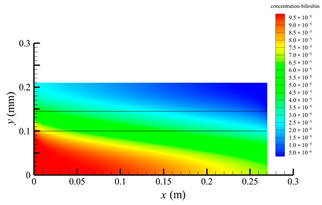	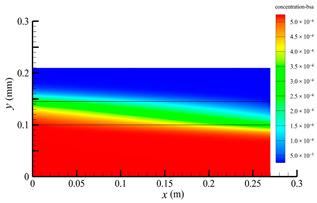
400	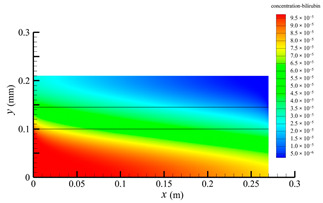	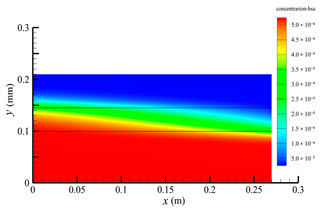
500	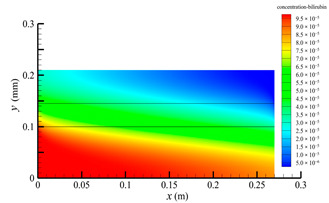	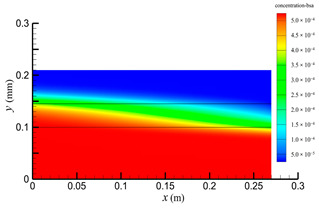
600	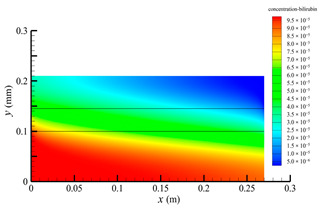	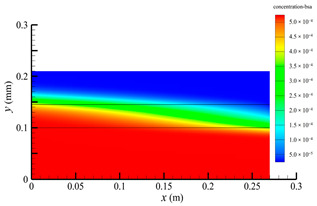

**Table 6:** The bilirubin and BSA concentration distributions for varying shell-side flow rates.

Shell-Side Flow Rate mL/min	Bilirubin	BSA
100	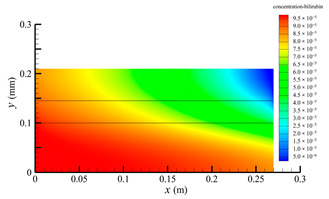	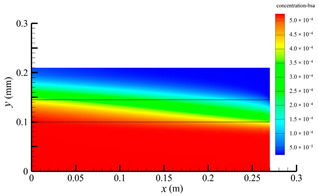
300	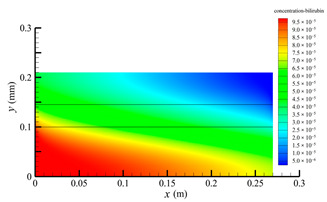	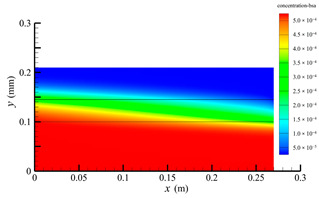
500	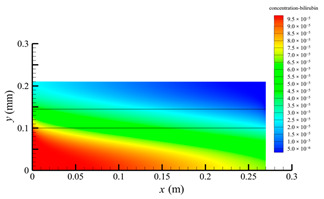	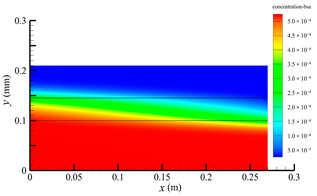
700	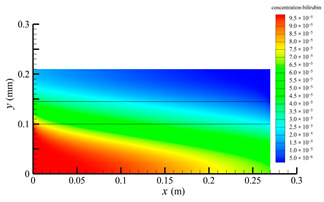	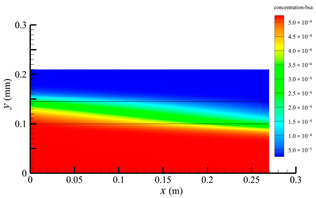
900	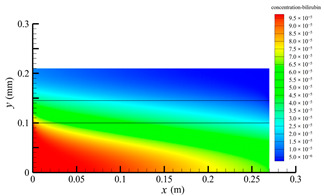	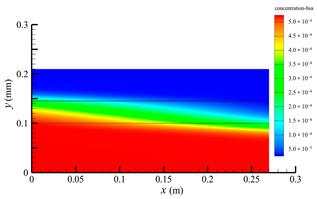

**Table 7:** The bilirubin and BSA concentration distributions for varying fiber tube lengths.

Fiber Lengths mm	Bilirubin	BSA
270	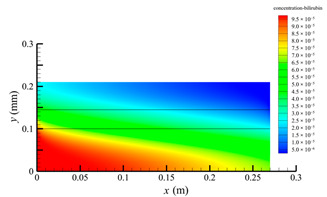	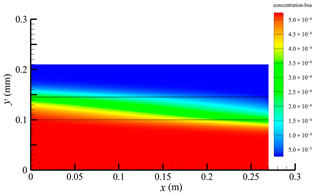
324	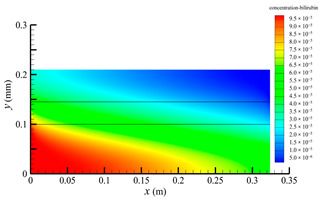	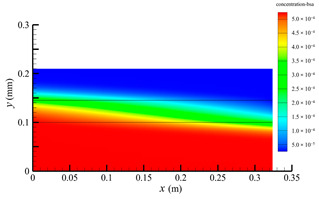
388.8	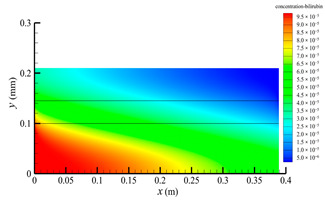	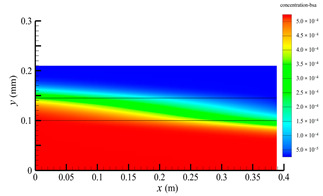
466.56	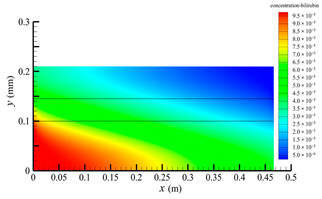	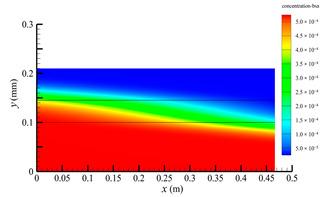
559.872	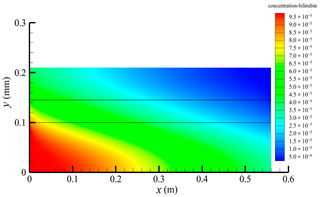	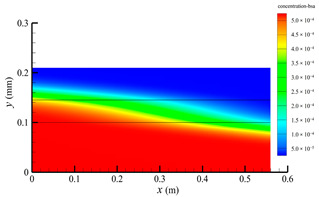

**Table 8:** Solute molecule characteristics, membrane pore size, and the corresponding mass transfer form.

Solute	Solute *MW* (Da)	$\mathrm{Solute}\;\mathrm{Stokes}\;\mathrm{Radius}\;r_{s}$ (nm)	Membrane Pore Size (nm)	Mass Transfer Form
Bilirubin	584.66	0.5	39.5	Convection (Dominated)
BSA	67,000	3.61	39.5	Diffusion & Convection

## Data Availability

The raw data supporting the conclusions of this article will be made available by the authors on request. The data are not publicly available due to ongoing researches using a part of the data.
